# Hybrid approach with convolutional neural network and regression models for age estimation in panoramic radiographs

**DOI:** 10.1007/s00414-026-03927-6

**Published:** 2026-07-29

**Authors:** Jhonatan Thiago Lacerda-Santos, José Alberto Souza Paulino, Hiarles Barreto Sampaio Brito, Fabiana Tolentino Almeida, Patrícia Meira Bento, Daniela Pita de Melo

**Affiliations:** 1 Department of Dentistry, State University of Paraíba, R. Baraúnas, 351, Bairro Universitário, Campina Grande, PB 58429-500 Brazil; 2 Computer Science PhD Student, Federal University of Campina Grande, Rua Aprígio Veloso, 882, Bairro Universitário, Campina Grande, PB 58429-900 Brazil; 3 Postgraduate in Oral and Maxillofacial Surgery, State University of Paraíba, R. Baraúnas, 351, Bairro Universitário, Campina Grande, PB 58429-500 Brazil; 4 College of Dentistry, University of Alberta, 11405 87 Ave NW, Edmonton, AB T6G 1Z1 Canada; 5 College of Dentistry, Department of Dentistry, State University of Paraíba, R. Baraúnas, 351, Bairro Universitário, Campina Grande, PB 58429-500 Brazil; 6 College of Dentistry, University of Saskatchewan, 105 Wiggins Rd, Saskatoon, SK S7N 5E5 Canada

**Keywords:** Age determination by teeth, Artificial intelligence, Computer Neural Networks, Decision trees, Panoramic radiography

## Abstract

**Background:**

Determining chronological age is a relevant aspect of criminal and civil interest for identifying corpses and refugees. This study aimed to estimate chronological age using a hybrid model that combines a convolutional neural network (CNN) with regression models based on panoramic radiographs.

**Methods:**

This observational, retrospective and cross-sectional study analyzed 2,546 panoramic radiographs from a Brazilian practice. Panoramic radiographs of patients over 18 years old with healthy, erupted lower premolars were selected. The radiographs were segmented using GIMP, and the dataset was divided into three sets: 70% for model training, 15% for validation, and 15% for testing. Based on the MobileNetV2 architecture, the CNN model extracted 576 attributes from each image, which were then used in conjunction with a decision-tree regression model to estimate age. To evaluate the model’s performance, metrics such as Mean Absolute Percentage Error (MAPE), Mean Absolute Error (MAE), Coefficient of Determination and Mean Square Error were used.

**Results:**

3,423 teeth were analyzed, and the patients’ mean age was 32.65 years. The overall MAE was 6.52 years (R² = 0.67); however, for the age group 31 to 35, the hybrid AI model had an MAE of 0.29 years for the male sample and 0.16 years for the female sample.

**Conclusions:**

The hybrid AI model proved accurate and robust for patients aged 31–35 years; however, in other age groups, the model performed unsatisfactorily, particularly in the 40–50 and 51–55 age ranges.

## Introduction

Radiographic images play a significant role in human age determination and are an essential tool in forensic science. Determining chronological age is of criminal and civil interest in identifying corpses and refugees [1–3]. Estimating the age of living individuals is important for situations involving asylum, employment, education, access to social services, and cases of rape, kidnapping, and human trafficking [4–6]. Thus, various dental assessment methods have been employed to determine an individual’s age [3, 7].

Teeth are the most resistant structures in the human body, and their morphology is preserved for longer than other organs. Therefore, they have been frequently used in studies for estimating age [1, 8]. Age estimation using teeth can be carried out by sectioning the tooth or through imaging exams [9]. Panoramic radiography is one of the most widely used imaging techniques for this purpose, particularly because it is a non-invasive modality that provides a comprehensive view of the jaw and dental development, which are key indicators of human development [3]. However, panoramic radiographs have limitations that can lead to errors in image interpretation due to anatomical overlap [10, 11]. To minimize subjective biases inherent in interpreting imaging exams due to human limitations, studies have utilized Artificial Intelligence (AI) to enhance the accuracy of lesion and degenerative disease diagnosis [12, 13].

In recent years, advances in AI techniques, particularly deep learning, have opened new perspectives on image analysis [12]. Convolutional Neural Networks (CNNs) have proven highly effective for image classification and prediction tasks due to their ability to extract and learn features directly from raw data [14–16]. Radiomics is a quantitative approach to imaging analysis. It aims to extract and quantify imaging features using deep-learning methods to enhance decision-making [17].

In age estimation, CNNs are promising tools for automating the process, overcoming the subjectivity inherent in traditional methods based on manual observations [18]. Recent studies demonstrate that these models can identify subtle patterns in panoramic radiographs that correlate with chronological age more accurately than experts. Furthermore, these techniques have the potential to reduce processing time and improve the reproducibility of results [19–22].

Although some studies have employed AI as an auxiliary method for age estimation using imaging [22–25], no method accurately estimates age across all age groups [24]. Thus, age estimation remains an open problem [6]. Therefore, this study proposed a hybrid AI model that uses a convolutional neural network for automated extraction of radiomic image attributes and a decision-tree regression model to estimate age from analysis of mandibular premolars in panoramic radiographs.

## Materials and methods

This observational, retrospective, cross-sectional study was approved by the local Research Ethics Committee (protocol number 6.700.058) and conducted in accordance with the Declaration of Helsinki. This study followed the Strengthening the Reporting of Observational Studies in Epidemiology (STROBE) guidelines.

### Dataset

The dataset was selected using the non-probability and convenience sampling method in accordance with the study’s eligibility criteria. 2,546 panoramic images were selected (1,369 from female participants and 1,177 from male participants), from the database of a Brazilian imaging center.

### Eligibility criteria

Panoramic radiographs of patients over 18 years of age, of both sexes, with healthy, fully erupted first or second lower premolars were included in the sample. Mandibular premolars were selected for this research due to their predominantly single-rooted characteristic and large pulp chamber. Previous studies have demonstrated positive results in age estimation using premolars with manual methods [4, 26–28]. Moreover, the projection of the spine in the anterior region on panoramic radiographs interferes with analysis of anterior teeth [26, 28], and the difficulty of finding samples of healthy molars in adults due to the high prevalence of tooth loss [2].

Excluded criteria were panoramic images with lower first or second premolars exhibiting any of the following conditions: presence of carious lesions; presence of a restoration; tooth with prosthetic rehabilitation (crown, bridge support, inlay, onlay, or overlay); impaction; tooth with an apicoectomy; tooth with endodontic treatment; tooth with an intraradicular post or core; dental rotations; hypercementosis; pulp calcification; external or internal root resorption; presence of associated cysts or tumors; fixed orthodontic appliance; and low-quality panoramic radiographs.

### Panoramic image segmentation

The images used in this research were taken with the Carestream^®^ CS 9000 3D panoramic device (Carestream Dental LLC, Atlanta, GA), which has a resolution of 3000 × 1750 pixels at 300 dpi, 24-bit intensity, 70 kV, and 10 mA.

The panoramic images were imported into the GNU Image Manipulation Program (GIMP^®^ 2.10.36) using the “File” option followed by “Open”. For segmentation, the “Free Selection Tool” was used, which allows the user to select specific regions of the image by cropping the selected area. An average of 40 points were selected and connected by a line that demarcated the area. To select the points, a 400x zoom was applied to achieve greater precision in the region chosen by the examiner.

With the delimitation of the tooth of interest, the “Crop” tool was used through the “Edit” option followed by “Cut” or through the keyboard shortcut “Ctrl + X”. Then, click on the “create a new image” option, and another window will open. In this new window, under the “model” item, the “A4 (300ppi)” size for the new image was selected, and the width and height were set to 400 pixels. In “advanced options” in the “color space” item, the “gray levels” option was selected and in the “fill with” item, the “front color” option was chosen.

**Fig. 1:**
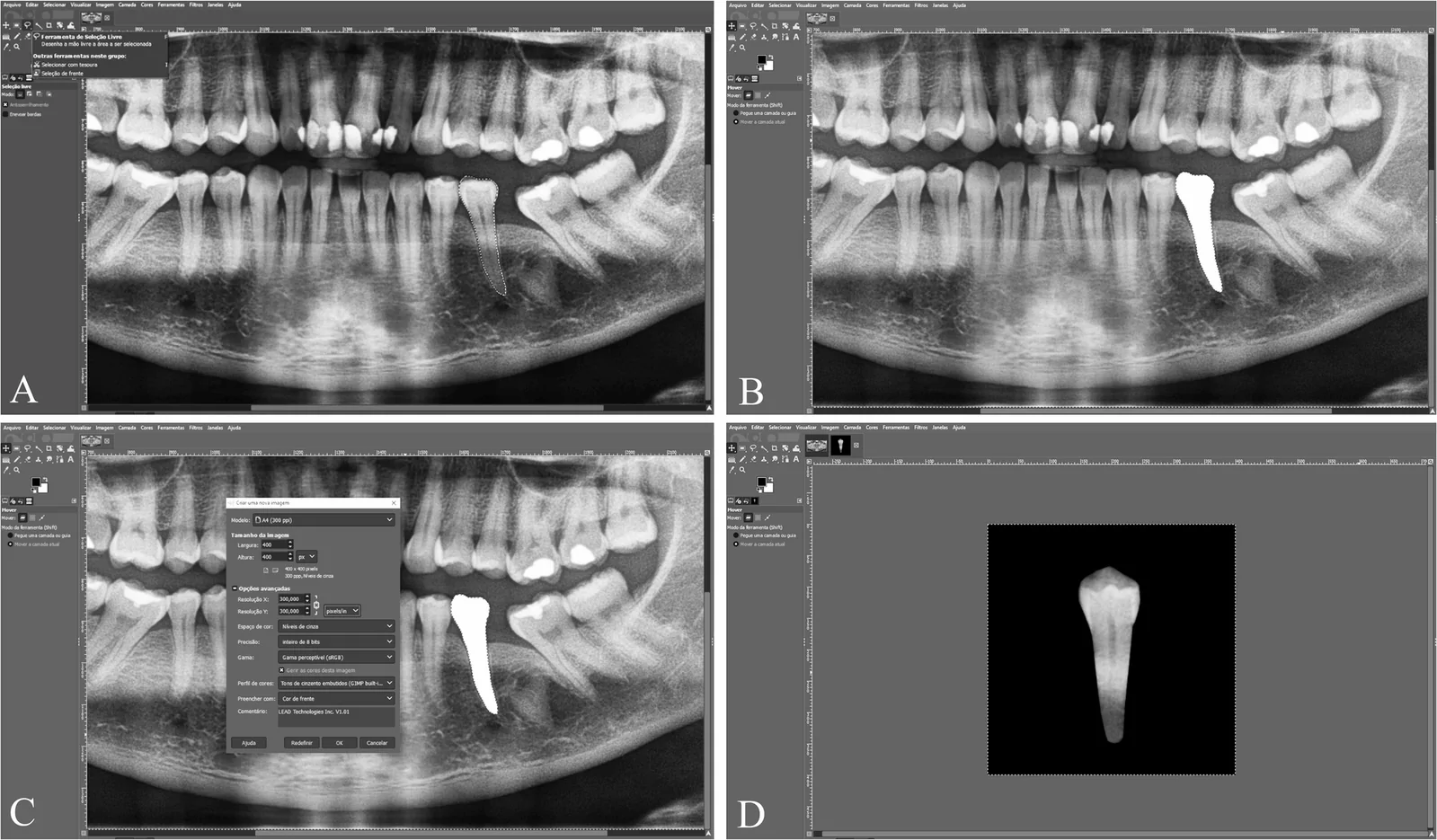
Image segmentation and saving process. **A**) GIMP software interface illustrating the tooth delimitation using the “free selection” tool. **B**) Cropped image of the tooth after segmentation of the panoramic radiograph. **C**) Parameters for the new layout used to paste the segmented tooth. **D**) Final image following the segmentation of the tooth

The segmented teeth were pasted into a new image with a black background, and the image was then exported in “jpg” format (Fig. 1). All saved images were coded according to the panoramic ID number and tooth number.

It should be noted that the segmentation process was performed manually by a single examiner with no prior knowledge of the patient’s sex or actual age. The examiner was a dentist with extensive professional experience in examining orthopantomograms. In addition, prior to data collection, the examiner carried out a study and training using panoramic images to improve the image segmentation method.

### Dataset preparation using AI

The sample was partitioned into three datasets: the first was intended for training the AI model (70% of the total sample), the second was reserved for validation (15% of the total sample) to evaluate model performance at each training iteration. Finally, the last part was destined for testing (15% of the total sample). This approach is known as cross-validation, in which the model was trained 5 times, with a different subset of training and validation data used each time. At the end, the average performance obtained in each round was calculated, representing the overall performance of our model. This method was used to minimize data waste, a significant advantage when sample size is small.

Due to an imbalance in the database, oversampling, also known as data augmentation, was performed to create synthetic data for the age groups with the fewest samples. This process was carried out only on the training set to avoid generating sampling bias on the test set. The oversampling technique adopted for this task is called SMOTE (Synthetic Minority Over-sampling Technique), which uses K-Nearest Neighbors (KNN) models to generate new samples based on the similarity of existing samples in the training set. SMOTE was applied using k = 3 nearest neighbors. This configuration was adopted to improve the diversity of synthetic samples while preserving local feature-space consistency for minority age groups.

### Extracting image attributes

To ensure reproducibility, feature extraction was performed using the MobileNetV3Small architecture available in TensorFlow/Keras with ImageNet pre-trained weights, enabling transfer learning from ImageNet features. The model was configured with an input shape of (224,224,3), indicating RGB input images with resolution 224 × 224 pixels and three-color channels. The original classification layer was removed, and a GlobalAveragePooling2D() layer was added to produce compact feature representations, yielding 576-dimensional embedding vectors for each image. The convolutional backbone was used exclusively as a fixed feature extractor (trainable=False), without fine-tuning.

Before feature extraction, all images were resized to 224 × 224 pixels and processed with tf.keras.applications.mobilenet_v3.preprocess_input, which applies the normalization scheme expected by the ImageNet-pre-trained model. The extracted features were standardized using StandardScaler library, which transforms the features to zero mean and unit variance. The scaler was fitted on the training set and subsequently applied to both training and testing embeddings. Finally, the standardized embeddings were used as input features for the XGBoost regression model that estimates chronological age.

**Fig. 2:**
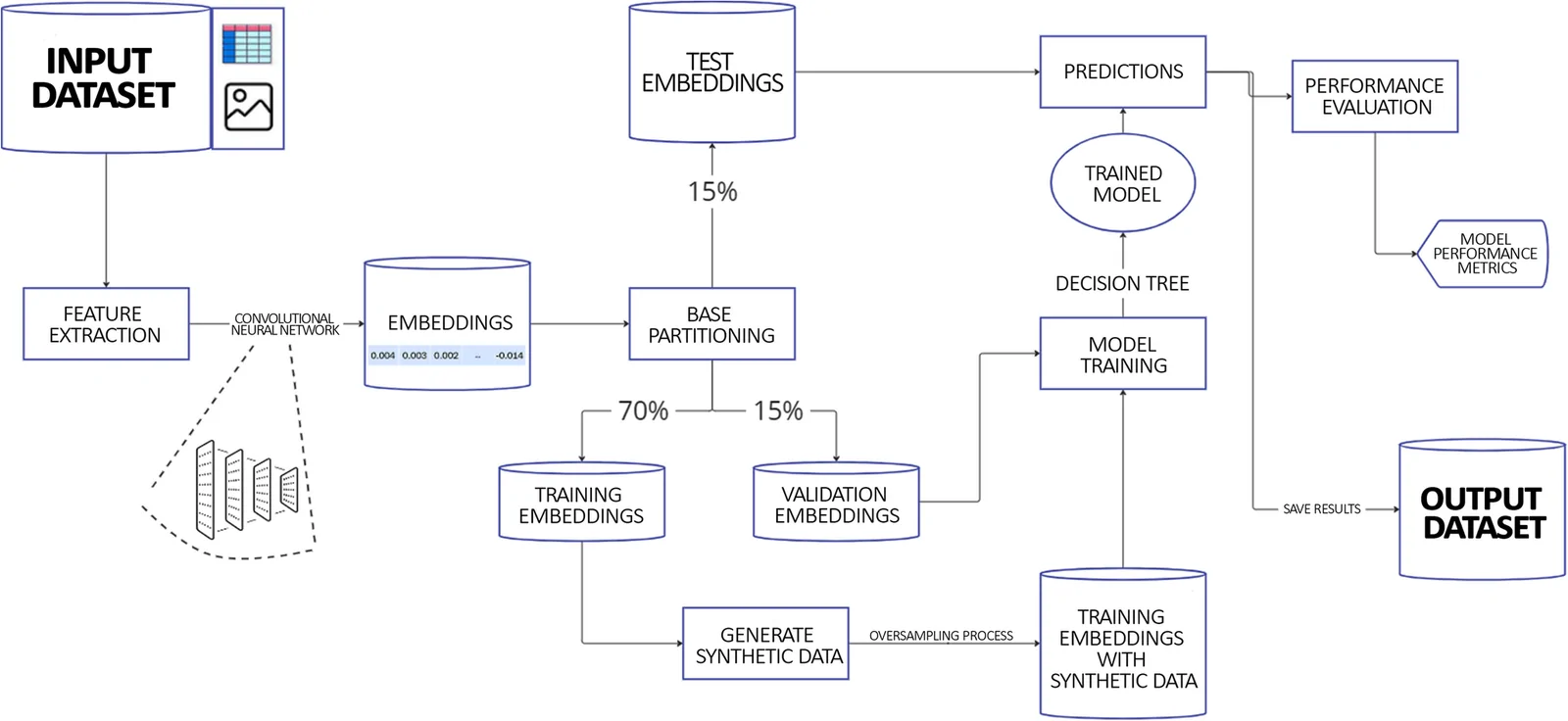
Flowchart used to construct the age estimator

In this sense, the summary of the data pipeline is indicated in the flow below and in Fig. 2.

Image Preprocessing: Segmented images were resized to 224 × 224 pixels using linear interpolation (cv2.INTER_LINEAR). Pixel values were normalized by dividing by 255.0, then the network-specific normalization scheme was applied using tf.keras.applications.mobilenet_v3.preprocess_input. CNN Parameterization: Feature extraction was performed using the MobileNetV3Small architecture initialized with ImageNet weights. The convolutional base was strictly frozen (trainable = False) to prevent fine-tuning and act purely as a feature extractor.

Feature Extraction: The original classification head was replaced with a GlobalAveragePooling2D() layer, producing a stable, compact 576-dimensional embedding vector for each image. Feature Scaling & Selection: The extracted embeddings were standardized to zero mean and unit variance using the StandardScaler library, fitted only on the training data and then applied to the test data. All 576 features were used as inputs for the regression phase without dimensionality reduction, relying on the inherent feature selection mechanisms of the Extremely Randomized Trees algorithm.

### Chronological age estimation method

Although individuals older than 18 years were eligible for the study, the final dataset effectively covered the age range of 20 to 55 years, based on the available radiographic samples after applying quality filtering and inclusion criteria.

As described in this methodology, two machine learning models were used to perform age estimation from radiographic images. The first corresponds to the CNN model mentioned in the previous topic, which was used to extract the embeddings. The second corresponds to a regression model based on decision trees that estimates age values using the embeddings extracted from each image. In this type of model, each sheet contains a numerical value that represents the prediction for that region of the feature space. Thus, the decision tree can predict the class label of each pixel in the image [29].

The MobileNetV2 network architecture consists of the following layers: one convolutional layer, followed by seven bottleneck layers; another convolutional layer, followed by a polling layer; and, finally, another convolutional layer [30].

### Metrics and statistical analysis

To analyze the performance of the proposed algorithm model, a widespread metric was applied to evaluate age estimation systems: the Mean Absolute Percentage Error, or Mean Absolute Percentage Error (MAPE), was used as a reference metric to choose the model with the best result. MAPE indicates, on average, how much the predictions differ from the actual values in percentage terms; that is, the lower the MAPE, the fewer mistakes the algorithm makes in estimating age [31].

As complementary metrics, to better evaluate the chosen model, the Mean Absolute Error (MAE) metrics were calculated: Mean absolute percentage error or Mean Absolute Percentage Error (MAPE), Coefficient of determination or R2 Score, and Mean Squared Error (MSE). Verification of CNN performance using the metrics module of the scikitlearn library (version 1.0.2).

## Results

**Fig. 3:**
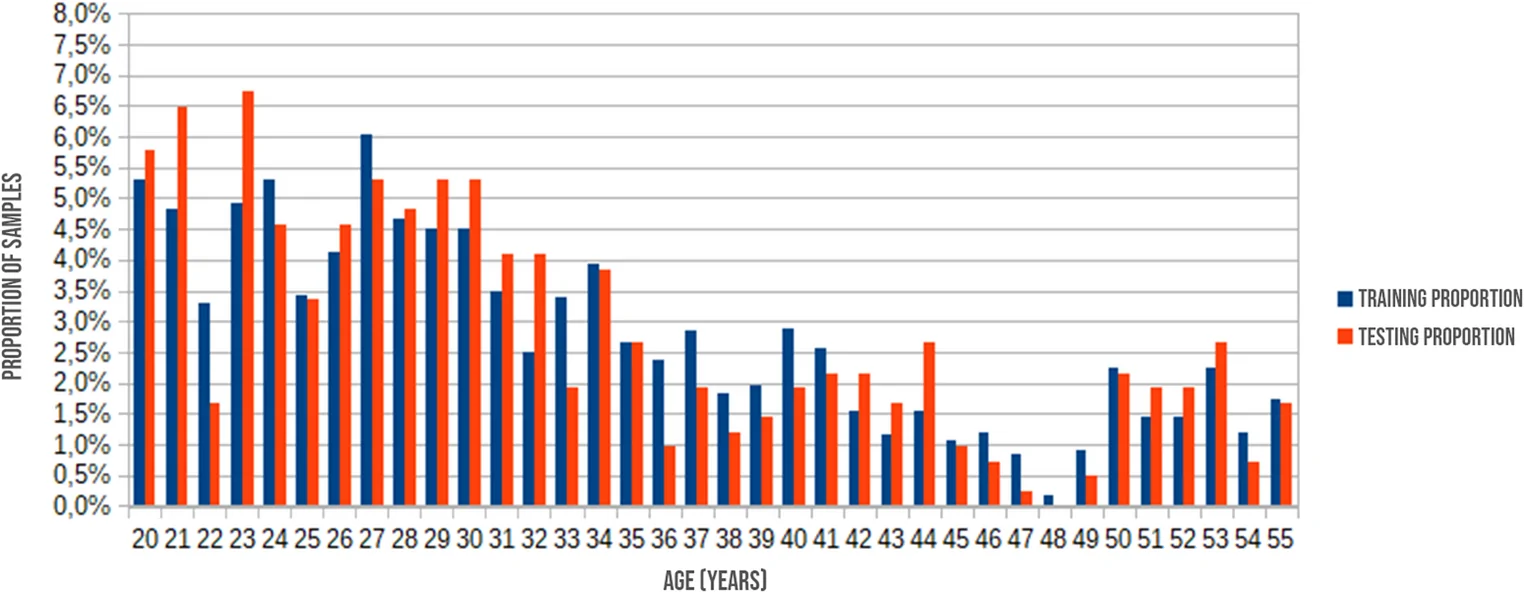
Distribution of age proportions by training and test base

A total of 3,423 mandibular premolar teeth were analyzed, divided into 1,900 first premolars and 1,523 s premolars. 1,369 female participants and 1,177 male participants were included, with a mean age of 32.65 years (SD ± 9.71) (Fig. 3).

The categorization of the ranges was conducted to analyze the model’s predictions and assess the results. Segmentation into age groups for training the developed model (using the complete training dataset) was not implemented. Therefore, the adoption of age groups was driven by the need to enhance the interpretation of the results and the model’s performance.

Regarding the chosen ranges, since no studies currently exist in the literature that perform a similar segmentation of predictions into age groups to serve as a reference for selecting these ranges, 5-year intervals were evaluated and empirically adopted. The distribution of the sample categorized by age group is outlined in Table 1.

**Table 1:** Sample distribution (images) by age group

Age group	Training/Validation	Tests	Total
20–25 years	639	119	758
26–30 years	561	105	666
31–35 years	377	69	446
36–40 years	280	31	311
41–45 years	184	40	224
46–50 years	126	15	141
51–55 years	190	37	227

### Training set

Nine regression models were evaluated, as described in Table 2, to select the model for age estimation based on CNN-extracted embeddings. The Extremely Trees Regressor model was found to be the best model, that is, the one with the lowest MAPE (12.1%), the smallest MAE (3.5 years), and the highest R2 Score (0.67).

**Table 2:** Results of the analyses of the regression models tested for age estimation

MODEL	MAPE	MAE (years)	R2	RMSLE
Extremely Trees Regressor	0.121	3.5026	0.6727	0.1625
Light Gradient Boosting Machine	0.1289	3.8293	0.6439	0.167
Extreme Gradient Boosting	0.1305	3.8617	0.6169	0.1726
Random Forest Regressor	0.1412	4.1846	0.6087	0.1752
K Neighbors Regressor	0.1607	4.5545	0.4289	0.2078
Gradient Boosting Regressor	0.1726	5.3271	0.4593	0.1988
Ridge Regression	0.1723	5.3686	0.4194	0.2093
Bayesian Ridge	0.1779	5.5983	0.4016	0.2059
Linear Regression	0.1782	5.4771	0.3495	0.2321

MAPE: Mean Absolute Percentage Error; MAE: Mean Absolute Error; R2: Coefficient of determination; RMSLE: Root Mean Squared Logarithmic Error

### Test set

The test set consisted of 382 images. The performance of the Extremely Trees Regressor model on this dataset was MAE 6.52, MAPE 0.23, R² Score 0.03, and MSE 60.98. The proposed model, which analyzed the entire database, demonstrated an MAE of 6.52 years.

**Fig. 4:**
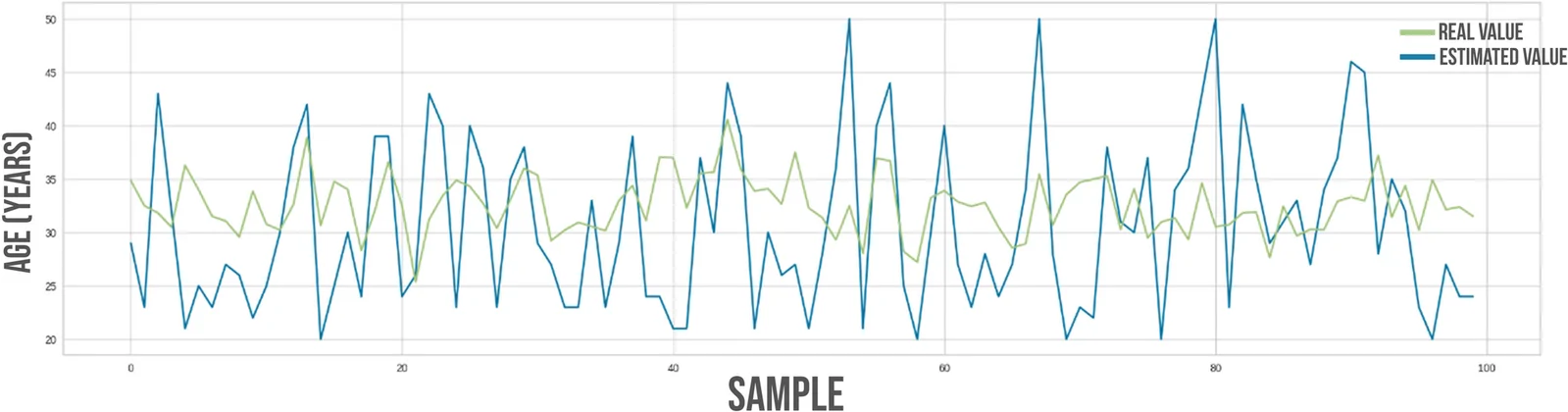
Comparison between the real chronological age (blue line) and the estimated age (green line) by the proposed model

When analyzing the sample by age group, the proposed model showed a lower MAE among individuals aged 31 to 35 years, corresponding to an error of only 0.29 years. However, the model was less accurate in estimating age for the 20–25, 41–45, 46–50, and 51–55 age groups, with MAEs of 9.23, 7.74, 12.78, and 17.42, respectively. Figure 4 compares the actual chronological age with the age estimated by the Extremely Trees Regressor algorithm.

### Results based on data from male participants

For the analysis of data from male participants only, the performance metrics of the proposed model were MAE: 6.24; MAPE: 0.21; R2 Score: 0.17; and MSE: 56.48.

**Fig. 5:**
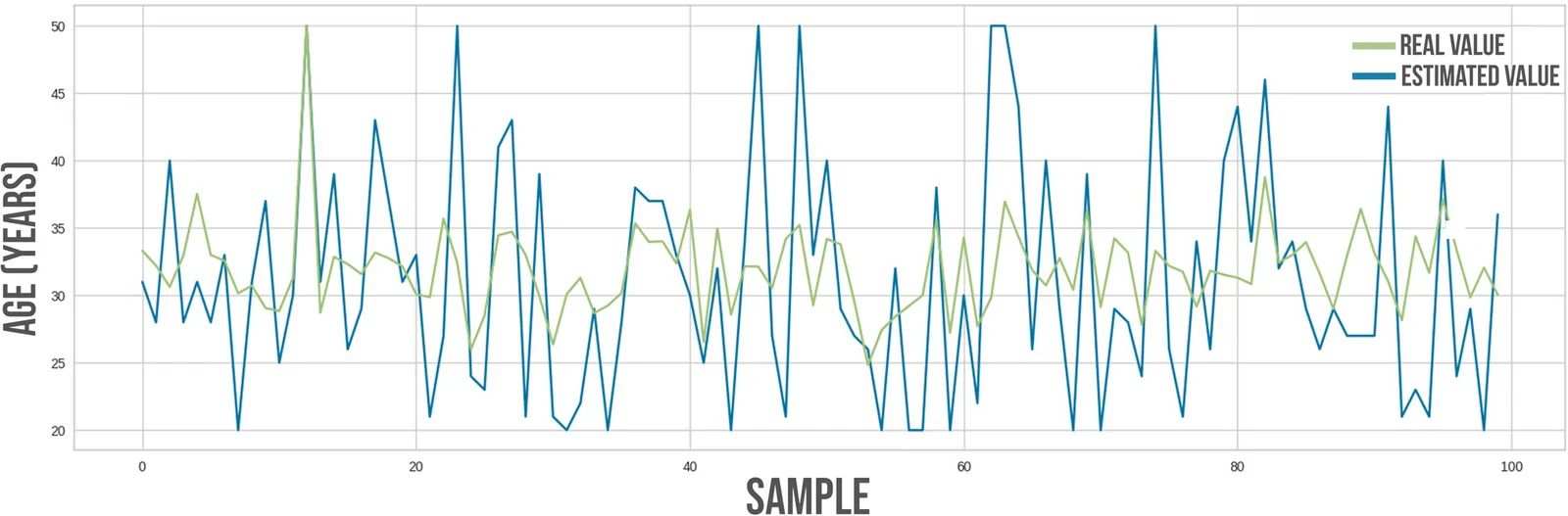
Comparison between the real chronological age (blue line) and the estimated age (green line) by the model proposed for male individuals

The analysis by age group of male participants found that the model estimated a lower MAE in individuals aged 31 to 35 years, corresponding to a difference of only 0.16 years. While the proposed model was less accurate in the age groups of 20–25 years (MAE: 8.82), 41–45 years (MAE: 8.7), 46–50 years (MAE: 11.67) and 51–55 years (MAE: 14.31). Figure 5 shows a comparison between the actual chronological age and the age estimated by the Extremely Trees Regressor algorithm, limited to male individuals.

### Results based on data from female participants

The metrics used to analyze data from female participants only were MAE: 6.17; MAPE: 0.21; R2 Score: 0.08; and MSE: 58.34.

**Fig. 6:**
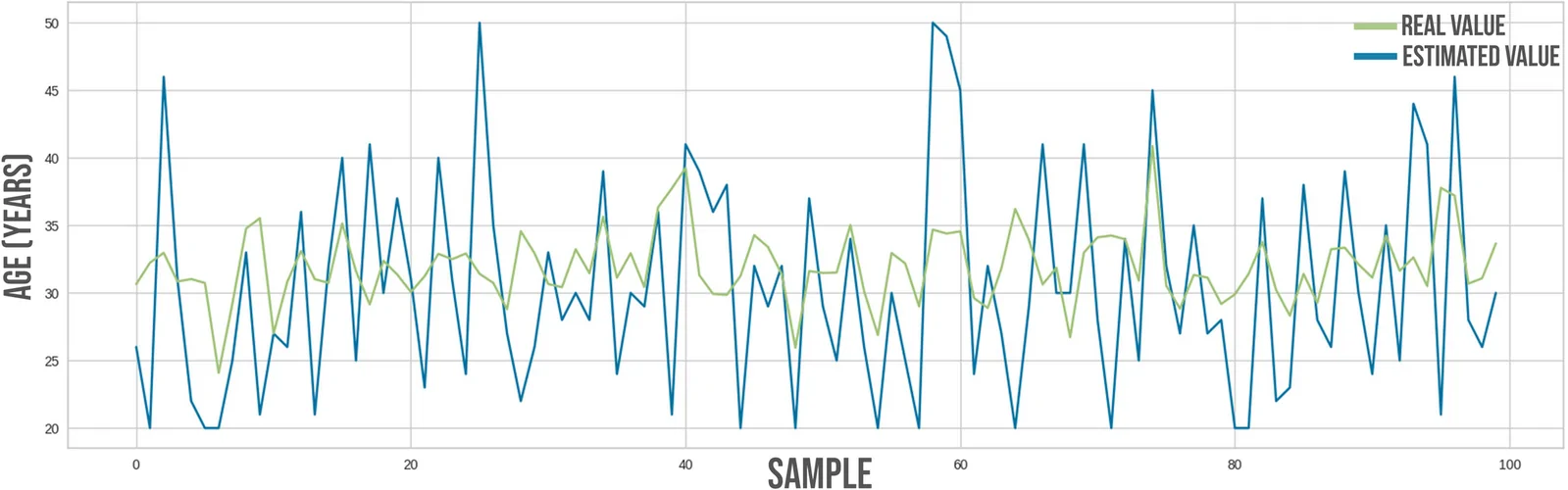
Comparison between the real chronological age (blue line) and the estimated age (green line) by the model proposed for female individuals

It was found that the lowest MAE in female participants was in the age group of 31 to 35 years, corresponding to 0.47 years. However, in the age groups of 20–25 years (MAE: 9.87), 41–45 years (MAE: 8.21), 46–50 years (MAE: 12.4) and 51–55 years (MAE: 16.64), the proposed model was less accurate. Figure 6 shows a comparison between the actual chronological age and the age estimated by the algorithm for female individuals.

## Discussion

The results of the present study were promising, combining a convolutional neural network for automated attribute extraction from radiological images with a tree-based model for attribute classification and age estimation using mandibular premolars in panoramic radiographs.

The most conservative form of assessment for estimating age using teeth consists of the use of imaging tests. In general, panoramic radiographs are widely used in age estimation through manual methods, such as those proposed by Demirjian, Willems, Cameriere and Nolla [4, 32–34]. The variability accepted by forensic medicine for age estimation based on dental analysis in adults is up to ± 10 years [7]. However, the application of manual methods in adult populations presents challenges, as they are more complex procedures that require examiner experience for accurate measurement, and they also require significantly longer execution times than automated methods [7, 35]. Thus, the use of automated methods, such as the one adopted in this research, contributes positively to the evaluation of characteristics and measurements obtained by computational means.

Automated methods for feature extraction, such as neural networks, enable a new approach to age estimation by automatically extracting relevant features from raw data without human intervention [36]. In general, the performance of CNNs is reported using the Mean Absolute Error (MAE), the standard measure for age estimation errors. In practice, the MAE is the absolute difference between the estimated and true ages [37]. Thus, a low MAE indicates that the estimated age is close to the true age.

In the present study, the regression model demonstrated satisfactory performance in estimating tooth age, with an MAE of 3.5 years (R² Score 0.67). Recent studies in forensic and dental imaging literature [38, 39] have reported comparable R² values for clinically acceptable models. For example, recent works in dental age estimation reported R² values around 0.63 while still considering the models suitable for practical application. Furthermore, chronological age prediction from dental images is inherently affected by substantial biological variability, anatomical heterogeneity, and population-specific aging patterns, which naturally limit the achievable linear correlation between predicted and actual ages. Despite these intrinsic limitations, the proposed approach achieved an MAE of approximately 3.5 years, indicating satisfactory predictive performance for the intended application.

The model proposed in the present study achieved an MAE of 6.52 years in a sample of individuals aged 20 to 55 years. Lower MAE values have been reported in previous studies assessing the Chinese population. For example, Fan et al. [40] analyzed 15,195 panoramic images of individuals aged 16 to 50 years using a deep learning model and reported an MAE of 2.61 years. A study conducted in Croatia analyzed 4,035 panoramic images of individuals aged 19 to 90 years, yielding an MAE of 2.95 years [41]. These differences can be explained by the specific dental characteristics of each population, sample size, and the variety of algorithms used in the cited research, which could influence the precision of age estimation. In the present study, the proposed model combined a CNN to extract image attributes with a regression algorithm based on decision trees to estimate age. This method can help reduce the time required to estimate age by automatically extracting image attributes.

However, the sample distribution is indeed non-uniform, with a strong concentration in younger groups. This imbalance introduces a risk of bias during model training, potentially favoring age groups with greater sample density. Consequently, the model was found to be less accurate in estimating age in the older groups with fewer samples, yielding a Mean Absolute Error (MAE) of 12.78 years for the 46–50 years group and 17.42 years for the 51–55 years group. Therefore, it is suggested that future research test the model on a sample with a uniform distribution by age group.

Another finding of the present study is that the proposed model achieves greater accuracy in the 31–35 age range, with an MAE of 0.29 years. However, this result should be interpreted with caution, as the chronological age prediction from dental images is inherently affected by substantial biological variability and anatomical heterogeneity, which naturally limit the achievable linear correlation. Furthermore, the low MAE in the 31–35 years subgroup may be a consequence of data distribution, favoring age groups with greater sample density.

Similarly, another investigation examined 10,782 radiographs from individuals aged 2 to 89, yielding an overall MAE of 3.26 years. In this study, the MAE varied significantly, with 1.48 years for the 2- to 9-year age group and 6.02 years for those aged 80 to 89 [42]. Additionally, when analyzing only the 30-39-year age group, an MAE of 3.16 years was found [42]. Therefore, the model’s accuracy for estimating age decreases as age increases. This aligns with the current study’s findings, as worse results were observed in the 40-50-year (MAE: 11.67) and 51-55-year (MAE: 14.31) age groups.

Despite the substantial sample sizes of the previously mentioned studies, the hybrid model used in the current study achieved a lower MAE for the age group in question. In addition to population differences, this discrepancy can be attributed to methodological differences: the previous studies used entire panoramic images, whereas the present study focused on segmenting specific teeth (mandibular premolars) for age estimation. This targeted approach may enhance accuracy, as relying on the entire image without segmentation allows the algorithm to extract features from the whole exam, potentially decreasing the model’s precision in age estimation.

Another promising result from the present study concerns the estimation of age by sex. In the 31–35 age range, the model achieved an MAE of 0.16 years for male participants and 0.47 years for female participants. While this difference exists, both results reflect highly accurate predictions within a margin of half a year. This variance might be attributed to the differing sample sizes of 1,369 female and 1,177 male participants in our dataset, as well as to inherent anatomical variations in tooth morphology that may present differently between the sexes. Some studies have used deep learning to estimate population sex using panoramic radiographs [36, 43, 44]. However, no studies were found that performed age estimation on a dataset separated by sex (female and male).

Previous studies have investigated age estimation methods in populations up to 18 years of age, presenting promising results for this age group [35, 45–47]. However, there is a clear need to explore other adult age groups due to the scarcity of published studies. Investigating populations over 18 years of age becomes relevant to assist unidentified individuals seeking asylum, work, school, and access to social services, as well as individuals who may be penalized due to crimes such as rape, kidnapping and human trafficking [4–6]. The scarcity of studies focused on populations over 18 years of age can be attributed to the difficulty in accurately estimating age in individuals over 25 years of age, since the complete development of permanent dentition reduces the characteristics available for analysis [41]. Corroborating this observation, studies using CNNs in older populations have shown that the performance of prediction models progressively decreases with age [23, 42, 48].

The limitations of the present study are related to the lack of standardization in sample size by age group, which may have compromised the variability of the data. This weakness occurred because obtaining panoramic examinations of older patients was difficult, given the low demand for dental care and the high prevalence of edentulism, which limited evaluation by dental groups. We also suggest further research with a multicenter approach that increases the database to cover several populations and, therefore, assess the effect of ethnicity on age estimation.

Despite the aforementioned limitations, this study has several strengths that underscore its relevance. Age estimation in a dataset of 20- to 55-year-olds has been little explored, and our study adds valuable insights to this area. In addition, a hybrid model was proposed that uses a CNN to automate the extraction of image attributes and a decision tree regression model to predict age. Notably, the proposed model showed high accuracy for the age range of 31–35 years, and, to the best of our knowledge, this is the first study to achieve greater accuracy in this population. Given this, the results are expected to contribute to forensic science by providing information on the age of people without legal identification, as well as to assist in the development of new research. New studies are needed to train and evaluate the proposed model in other population groups with different social contexts, as well as to evaluate its performance in other dental groups.

## Conclusion

The proposed model demonstrated robust estimation only among patients aged 31 to 35 years. In other age groups, the model showed unsatisfactory performance, particularly in the 40–50 and 51–55 age ranges.
